# Supplementary material for: Flavin Binding to the Deca-heme Cytochrome MtrC: Insights from Computational Molecular Simulation
Source: Biophys J. 2015 Dec 15;109(12):2614–24. doi: 10.1016/j.bpj.2015.10.038 (PMC4699859; doi:10.1016/j.bpj.2015.10.038)
Supplement: Document S1. Supporting Materials and Methods, one figure, and one table [file mmc1.pdf]

Flavin binding to the deca-heme cytochrome MtrC: Insights from computational molecular simulation

M. Breuer, K. M. Rosso, J. Blumberger

October 20, 2015

Supporting Information

1 Docking to MtrC in SH state

In the following we would like to discuss our docking studies to MtrC in the SH state in more detail. As outlined in Section 2 in the main text, four docking regions were chosen for docking FMN in the SH state. This was motivated by the observed large switching motion of the loop: This not only changed the structure around the area of the original disulphide bond (i. e. around Cys444, see Figure 7A in the main text) but also led to the localization of Cys453 and its adjacent residues at the front of the protein as illustrated in Figure 7B, main text. Thus, both of these areas were regions of interest. In addition, we noticed that the loop segment between the two cysteines (residues 445 to 452) now passed by in the proximity of heme 4 and 5. These were the only hemes whose local environment was visibly changed by the conformational switch and thus this was a region of particular interest for docking. Finally, we also included the region around heme 7 which had been suggested in experiment as a binding site in the SH state.[1]

The initial scans of 300 runs for all 20 dockings (the above four regions for five different snapshots) yielded no affinities stronger than the 490 μM found for heme 2 in the SS state, nor any other interesting features in most cases. However, we decided to investigate two dockings further, one for heme 4 and one for heme 7. The docking for heme 7 was studied further as it had shown the overall strongest affinity (760 μM); and the docking for heme 4, while having shown a weaker affinity of 2.2 mM, yielded a docking site that had actually formed between the proprionates of hemes 4 and 5 on the one hand and different residues of the cys-loop on the other hand - the structure presented in Figure 7C in the main text. Hence we carried out another 900 runs in each case, subsequently clustering the 1200 runs in total at an RMSD cutoff of 3.0 Å as for the SS dockings. In the following we discuss the results for both of the hemes.

For heme 4, the clustering of all 1200 runs at 3.0 Å revealed two best poses of equal affinity (both 2.2 mM): The aforementioned pose and a similar pose where the flavin head group is rotated a bit. In a coarser reclustering at 4.0 Å these two clusters actually merge and yield a large first cluster (116/1200 runs) which is succeeded in size only by a cluster worse in binding free energy by 0.63 kcal/mol (containing 168 runs). This stands in contrast to the observations for docking to heme 2 in the SS state which yielded a much more ambiguous picture with the energetically best cluster containing very few runs only (see the histogram in Figure 5; the qualitative features do not change when reclustering at 4.0 Å). This suggests that this best cluster obtained by docking to a snapshot from the annealing trajectory is more significant (compared to the other runs from that docking) than the best pose shown for heme 2 in Figure 6B. (Albeit the picture is still not nearly as clear as for the FMN-binding protein redockings in Figure 4.) In the following, we will inspect this binding pose more closely. Figure 7C in the main text shows the close-up of this best cluster and the hydrogen bonds involved.

While this FMN pose forms only seven hydrogen bonds (compared to eight for the best pose at heme 2 in the SS state), two of them are formed by the head group, involving H-bond donors from different directions: The

amino group of Lys465 and the backbone of Lys449, both part to the cys-loop. Lys465 actually forms an ionic bond with a proprionate of heme 4 which appears to be quite stable. (Suggesting that the proximity and hence availability of this lysine is not just coincidence.) The interactions of the tail involve three hydrogen bonds of the hydroxy-hydrogens to the proprionates of hemes 4 and 5; and an ionic interaction between the phosphate and Lys449, as well as hydrogen bonds between the phosphate and the same lysine and the backbone of Gly448 (also part of the cys-loop). Thus, while the tail can always be expected to find some hydrogen bonding partners (see our "negative test" docking to a random region at the bottom of Domain I), in this case the interactions happen with two different parts of the protein: The hemes from Domain II on the left side and different residues from the cys-loop on the right, which in particular hydrogen-bonds with two of the four potential hydrogen-bonding sites in the head group. The edge-to-edge distances of this pose are 5.2 Å to heme 5 and 7.1 Å to heme 4, in principle still close enough for relevant ET rates. It should also be noted that this is just one out of five snapshots investigated from the annealing trajectory; chances are that the protein has still not found its final structure after 110 ns or that induced fit effects might yield stronger actual interaction with the docked flavin. (Apart from the obvious possibility of Lys449, which ion-binds the phosphate, optimizing its conformation, further hydrogen bonds might be formed between one of the remaining potential acceptors in the flavin head group and some other backbone hydrogen from the cys-loop.) Hence, while we could not find a binding site yet that would yield affinities in agreement with the stable binding observed by Edwards et al.,[1] we would argue that the pose we found close to hemes 4 and 5 suggests a potential binding site as: the flavin head group actually enters some form of cleft, rather than just lying somewhere on the protein surface; in doing so, it forms several hydrogen bonds with comparatively buried partners; the tail simultaneously interacts with both sides of the cleft - the hemes on the left and the cys-loop on the right/top; and the formation of this site depends on the conformational change we observed, that brought the cys-loop into the position to form this cleft. Further investigations could take off from this tentative binding pose and aim at identifying an actually strong binding site.

For heme 7, we obtained a strongest affinity of 530 μM over the 1200 runs and the best pose was clearly separated from all following clusters by an energy difference of -1.21 kcal/mol. However, with an edge-to-edge distance to heme 7 of 9.9 Å, this binding pose seems hardly relevant for ET. In addition, even though this pose did form two hydrogen bonds involving the head group, all interacting protein residues were already closeby and to some degree solvent-exposed in the crystal structure, and none of them was part of the cys-loop; therefore, the occurence of this docking site does not seem connected to the conformational change of the cys-loop.

As an aside, in order to investigate the impact of the redox state, we repeated these 1200 dockings for both heme 4 and 7 with all heme charges set to oxidized and clustered again at 3.0 Å RMSD cutoff. **(While the simulated annealing had been carried out in the all-oxidized state to match the experimental redox state,[1] all dockings described above were carried out in the all-reduced state for comparison with**

results in the SS state; see Materials and Methods in the main text.) For heme 4, the first two poses were qualitatively unchanged and their binding free energies merely changed by -0.4 and -0.3 kcal/mol, respectively. We checked the next few poses and could also find them (with small changes) among the first clusters of the previous all-reduced dockings, suggesting that the same general poses were found and mainly changed their energetical ordering. The observations were similar for heme 7 where the best pose showed minor changes and changed in binding free energy by -0.5 kcal/mol; and the next few poses obtained with oxidized hemes could also be found among the poses obtained with the reduced heme charges, again with different ordering. These results suggest that our docking results are rather insensitive to the exact heme redox state (for a given protein configuration), with the main effect a slight increase in affinity.

Table S1: RESP charges used for flavin mononucleotide (FMN) as obtained with the B3LYP functional[2,3] and cc-pVTZ basis set, combined with the COSMO continuum solvation model[4] **using a relative permittivity of 4.0**.

Atom name	Charge [e]	Atom name	Charge [e]
N1	-0.396715	C5'	0.069370
C2	0.373383	O5'	-0.411130
O2	-0.560608	P	0.923193
N3	-0.040698	O1P	-0.763397
C4	0.149203	O2P	-0.763397
O4	-0.517263	O3P	-0.763397
C4A	0.590519	H3	0.237377
N5	-0.544057	H6	0.125184
C5A	0.162506	H7M1	0.083170
C6	-0.134921	H7M2	0.083170
C7	0.029809	H7M3	0.083170
C7M	-0.240263	H8M1	0.047064
C8	0.069789	H8M2	0.047064
C8M	-0.081776	H8M3	0.047064
C9	-0.180532	H9	0.116942
C9A	0.016667	H1'1	0.037677
N10	0.076287	H1'2	0.037677
C10	0.050082	H2'	0.098083
C1'	0.091311	HO2	0.357665
C2'	-0.022327	H3'	0.074538
O2'	-0.542320	HO3	0.253008
C3'	0.015111	H4'	0.115456
O3'	-0.366671	HO4	0.380892
C4'	0.051154	H5'1	0.031974
O4'	-0.628058	H5'2	0.031974

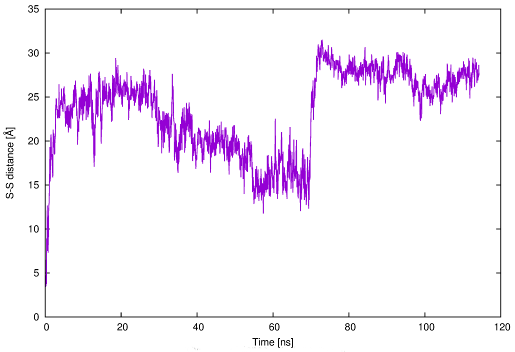

Figure S1: The S-S distance between Cys444 and Cys453 over the course of Simulated Annealing and subsequent dynamics at room temperature.

References

[1] Edwards, M. J., G. F. White, M. Norman, A. Tome-Fernandez, E. Ainsworth, L. Shi, J. K. Fredrickson, J. M. Zachara, J. N. Butt, D. J. Richardson, and T. A. Clarke. 2015. Redox linked flavin sites in extracellular decaheme proteins involved in microbe-mineral electron transfer. *Sci. Rep.* 5:11677.

[2] Becke, A. 1993. Density-functional thermochemistry. iii. the role of exact exchange. *J. Chem. Phys.* 98:5648.

[3] Lee, C., W. Yang, and R. Parr. 1988. Development of the colle-salvetti correlation-energy formula into a functional of the electron density. *Phys. Rev. B.* 37:785.

[4] Klamt, A., and G. Schuurmann. 1993. Cosmo: a new approach to dielectric screening in solvents with explicit expressions for the screening energy and its gradient. *J. Chem. Soc. Perkin Trans.* 2:799.
