# Supplementary material for: Flavin Binding to the Deca-heme Cytochrome MtrC: Insights from Computational Molecular Simulation
Source: Biophys J. 2015 Dec 15;109(12):2614–24. doi: 10.1016/j.bpj.2015.10.038 (PMC4699859; doi:10.1016/j.bpj.2015.10.038)
Supplement: Document S2. Article plus Supporting Material [file mmc2.pdf]

Article

Flavin Binding to the Deca-heme Cytochrome MtrC: Insights from Computational Molecular Simulation

Marian Breuer,[1] Kevin M. Rosso,[2] and Jochen Blumberger[1,*]
[1]University College London, London, United Kingdom; and [2]Pacific Northwest National Laboratory, Richland, Washington

ABSTRACT Certain dissimilatory bacteria have the remarkable ability to use extracellular metal oxide minerals instead of oxygen as terminal electron sinks, using a process known as “extracellular respiration”. Specialized multiheme cytochromes located on the outer membrane of the microbe were shown to be crucial for electron transfer from the cell surface to the mineral. This process is facilitated by soluble, biogenic flavins secreted by the organism for the purpose of acting as an electron shuttle. However, their interactions with the outer-membrane cytochromes are not established on a molecular scale. Here, we study the interaction between the outer-membrane deca-heme cytochrome MtrC from *Shewanella oneidensis* and flavin mononucleotide (FMN in fully oxidized quinone form) using computational docking. We find that interaction of FMN with MtrC is significantly weaker than with known FMN-binding proteins, but identify a mildly preferred interaction site close to heme 2 with a dissociation constant (K_d) = 490 μM, in good agreement with recent experimental estimates, K_d = 255 μM. The weak interaction with MtrC can be qualitatively explained by the smaller number of hydrogen bonds that the planar headgroup of FMN can form with this protein compared to FMN-binding proteins. Molecular dynamics simulation gives indications for a possible conformational switch upon cleavage of the disulphide bond of MtrC, but without concomitant increase in binding affinities according to this docking study. Overall, our results suggest that binding of FMN to MtrC is reversible and not highly specific, which may be consistent with a role as redox shuttle that facilitates extracellular respiration.

INTRODUCTION

Dissimilatory metal-reducing bacteria like *Shewanella oneidensis* possess the remarkable ability to utilize solid, extracellular metal oxides as terminal electron acceptors in place of oxygen. This unusual respiratory ability is facilitated by extracellular electron transfer (EET) between multiheme *c*-type cytochromes, located on the outer membrane (OM) of these bacteria (OM cytochromes) (1,2), and the metal oxide (see (3,41) for recent reviews). Several mechanisms have been found to be relevant for EET: the OM cytochromes can transfer electrons to solid substrates either by direct contact (4) or via biogenic, soluble redox shuttles secreted by the organism, in particular flavins (5). Furthermore, EET can take place directly at the cell surface or via micrometer-long conductive appendages, often termed “bacterial nanowires”. The latter have been recently shown to be OM cytochrome-containing extensions of the outer membrane (6). The relative physiological importance of these different mechanisms, direct versus shuttle and cell surface versus appendage-mediated, is still unclear.

While many aspects of flavin binding to OM cytochromes have been established (7–9), the molecular details of the binding interactions are still obscure: OM cytochromes typically contain several hemes (10 in MtrF (10) and MtrC (11), 11 in UndA (12)) and it is not known whether binding is unspecific, or specific to one or a few hemes.

For the cytochromes whose x-ray structures could be determined, cocrystallization with flavin ligands was not possible (10,12). By contrast, recent whole-cell voltammetric measurements suggested that flavins can stably bind to OM cytochromes (13). But this observation seems at odds with nuclear magnetic resonance (NMR) measurements (14) that report relatively high dissociation constants (K_d) of ~30–250 μM for flavin mononucleotide (FMN) to OM cytochromes, indicating weak and transient binding suitable for a role as electron shuttle.

A possible explanation of the contradicting experimental results was recently offered by Edwards et al. (11) The authors report a qualitative change in affinity of riboflavin or FMN to the model OM cytochrome MtrC from *S. oneidensis* upon reduction of a disulphide bond in the protein. While neither flavin stably binds to MtrC when the disulphide bond is intact (SS state), both flavins stably associate with MtrC once this bond is cleaved (SH state). The authors suggest that the apparently contradictory previous observations may be reconciled by their finding: the NMR measurements of Okamoto et al. (13) were carried out under ambient and hence possibly sufficiently oxidative conditions (retaining the SS state and low binding affinity), whereas the voltammetric measurements of Paquete and Louro (14) could have allowed for the bond to be cleaved,

Submitted June 23, 2015, and accepted for publication October 29, 2015.
*Correspondence: j.blumberger@ucl.ac.uk
This is an open access article under the CC BY license (http://creativecommons.org/licenses/by/4.0/).
Editor: Michele Vendruscolo.
© 2015 The Authors
0006-3495/15/12/2614/11

http://dx.doi.org/10.1016/j.bpj.2015.10.038

forming the SH state with a high binding affinity. Specific molecular insights into possible flavin binding site(s) are not available, however. In particular, it remains unclear whether the increased binding affinity in the SH state implies that the flavin binding site(s) are close by the disulphide bond or if the disulphide bond reduction triggers a long-range conformational protein transition, resulting in an increase of binding affinity at a site remote from the disulphide bond.

In this study, we would like to add a computational perspective to the ongoing questions on the nature of flavin binding to OM cytochromes, complementing our previous computational work on intraprotein electron transfer in these proteins (15–17). Here we focus on MtrC whose crystal structure has only very recently been reported in Edwards et al. (11). The protein consists of four domains: two penta-heme domains consisting of α-helices containing the CXXCH heme binding motifs, and two domains consisting of a large β-barrel each, with domain III containing the above-mentioned disulphide bond (see Fig. 1 *A*). The hemes are arranged in a staggered-cross pattern, with an octa-heme chain running along the length of a protein, sharing two hemes with a perpendicular intersecting tetra-heme chain.

To investigate flavin binding, we performed extensive docking studies of FMN to MtrC in the SS state for which the crystal structure was recently solved (11). The molecular

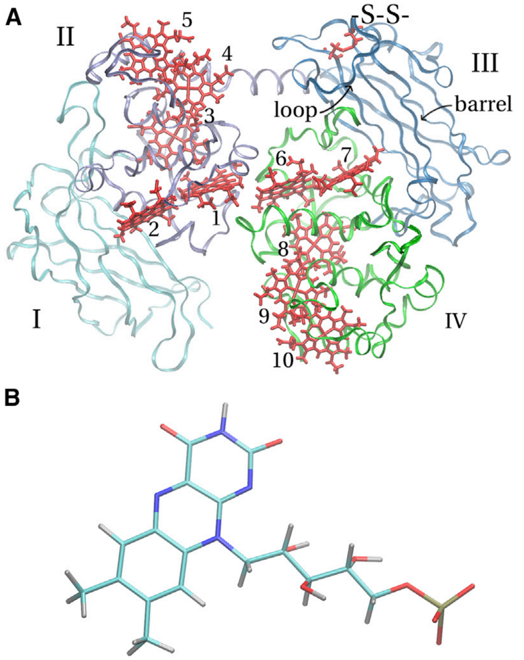

FIGURE 1 (*A*) The crystal structure of MtrC (11). Roman numerals depict the four domains, Arabic numerals denote the 10 heme cofactors. Domain III contains a barrel (labeled) suggested to be relevant for FMN binding (11). The labeled loop on the top of Domain III contains the disulphide bond (*red*, labeled as -S-S-). (*B*) The molecular structure of FMN. (*Cyan*) Carbon; (*blue*) nitrogen; (*red*) oxygen; (*silver*) hydrogen. The tricyclic headgroup is the redox-active moiety. To see this figure in color, go online.

structure of FMN is shown in Fig. 1 *B*: it consists of a redox-active, anthracene-derived heterocyclic headgroup; an aliphatic ribitol side chain; and a monophosphate tail. At the absence of any concrete experimental evidence regarding possible docking sites, we treat all except two buried hemes (8 and 3) as well as the vicinity of the disulphide bond as potential docking sites. We employ a blind-docking protocol in combination with a genetic algorithm, as previously suggested by Hetényi and van der Spoel (18,19). After validation of the method against known FMN-binding proteins, the protocol is used to dock FMN in the entire space of large predefined search regions around each heme of MtrC. We have also carried out simulated annealing (SA) molecular dynamics (MD) simulations to generate possible protein structures in the SH reduced form, for which no experimental structure is available to date. During the work leading to this article, a related docking study was reported for three homologs of MtrC (14). Here we focus on the unstudied MtrC and report on a refined docking protocol that allows us to study possible binding motifs to each heme in full atomistic detail.

Anticipating our results for known FMN binding proteins, we find that docking poses and binding affinities could be obtained in good agreement with experiment. Our results for MtrC in the SS form suggest that there is no single strongly preferred interaction site for FMN binding. However, we found a mild preference for FMN binding to one particular heme (heme 2). The best docking pose in the vicinity of heme 2 gives a dissociation constant $K_d =$ 490 μM, in very good agreement with the experimentally determined value, 255 μM (14). This is orders-of-magnitude higher than for typical FMN-binding proteins and supports the view that binding to MtrC is rather weak. This is traced back to the fewer number of hydrogen bonds that the headgroup of FMN can form with MtrC compared to FMN-binding proteins. MD simulation in the SH reduced form resulted in a conformational change of a loop on top of the β-barrel in Domain III (see Fig. 1 *A*), which is restrained by the closed disulphide bond in the SS state. A new binding motif at hemes 4 and 5 was identified for this conformation, although with still relatively weak binding affinities.

In the following section, the protocol used for docking of FMN to two flavin-binding proteins and MtrC is described as well as the protocol for MD simulation of MtrC. In Results and Discussion, the docking protocol is validated by redocking of FMN to two flavin-binding proteins with known binding site and binding affinity. Then, the results for docking of FMN to the SS form of MtrC are presented. They are compared to available experimental data and to the results obtained for the flavin-binding proteins. Finally, the conformational change of MtrC upon cleavage of the disulphide bond as obtained from MD simulations is described and the results of docking of FMN to the new conformation are discussed. The article is then concluded

with a comment on the functional relevance of the results reported.

MATERIALS AND METHODS

Redocking to flavin-binding proteins

The blind docking protocol established by Hetényi and van den Spoel (18,19) was tested on a number of different ligands, but not on flavins. Hence we decided to validate the method by redocking FMN to the flavin-binding protein (FMN-bp) from *Desulfovibrio vulgaris* (Miyazaki F) (PDB: 1AXJ) (20) and to NAD(P)H:acceptor Oxidoreductase (FerB) from *Paracoccus denitrificans* (PDB: 3U7R) (21). For both proteins the structure of the protein-FMN complex and experimental binding affinities are available, enabling a validation of the method. The NMR structure for FMN-bp contains 20 configurations; configuration 5 was chosen for the redocking. The crystal structure of FerB is dimeric; we redocked to the FMN binding site in monomer B but used the entire dimer structure during docking. The bound FMN structure was removed from both proteins; in the case of FerB, crystal water, and one nonaethylene glycol were stripped as well and one selenomethionine was mutated to a regular methionine as no parameters were available for selenium. Furthermore, protonation states for ionizable groups in FerB were set according to the results of the pK_a estimator PropKa 3.1 (22). For FMN-bp, the NMR structure already included hydrogens. The protein coordinates themselves were used as-is without force-field relaxation/minimization. However, to optimally place the search box (see below) around the protein, FMN-bp was rotated by 20° around the *z* axis, −30° around the *x* axis, and 10° around the *y* axis. Before any dockings, nonpolar hydrogens in both FMN and proteins were merged with their parent carbons using the utility program AutodockTools.

Ligand docking was carried out using Autodock 4.2 (23), which enables conformational searches of docking poses by a number of search algorithms and provides binding free energies based on an empirical free energy force field (23–25). The default atomic parameters in Autodock were used except for the atomic charges, which were taken from the AMBER03 force field (26). Atomic charges for FMN were obtained according to the RESP procedure (27) from a discrete Fourier transform electronic structure calculation (using NWChem (28)) with the B3LYP exchange correlation functional (29,30) and cc-pVTZ basis set, combined with the Cosmo continuum solvation model (31). We used a relative permittivity ϵ_r of 4.0 to describe the low-permittivity environment of the protein; however, even the opposite extreme of a bulk water environment ($\epsilon_r = 78.4$) yielded RESP charges that differed by <0.03 unit charges for each atom and by 0.01 unit charges on average. Hence, we concluded that the charge parametrization is insensitive to the assumed dielectric environment. The atomic charges for FMN with $\epsilon_r = 4.0$ are summarized in Table S1 in the Supporting Material. During docking, intramolecular electrostatic interactions and hydrogen-bonding terms between atoms of FMN were switched off. This was found to be necessary to prevent spurious formation of intramolecular hydrogen bonds between the ribitol hydroxy groups and the phosphate group of FMN. As we will see in Results and Discussion, the redocking of FMN to the two flavin-binding proteins with the same intramolecular interactions switched off were rather successful, justifying this ad hoc approach.

The blind docking was carried out employing the Lamarckian genetic algorithm as implemented in Autodock. This is a modified genetic algorithm featuring occasional local optimizations of individuals. All parameters relating to the genetic algorithm were kept at their default values except as mentioned otherwise. For FerB, a 35 × 37 × 27 $Å^3$ search box with 96 × 98 × 72 grid points was used, centered at 19.0, −5.0, and 69.5 Å, and for FMN-bp, a 23 × 38 × 38 $Å^3$ search box with 62 × 100 × 100 grid points was used, centered at 8.0, 2.0, and 3.0 Å (see Fig. 2, *A* and *C*). These boxes were chosen to resemble the search conditions for MtrC (see further below) and were large enough to cover most of the protein surface of FMN-bp and a large surface on the bigger protein FerB, hence allowing for the flavin to probe protein regions far away from the experimentally determined binding site. For each protein, 1200 docking

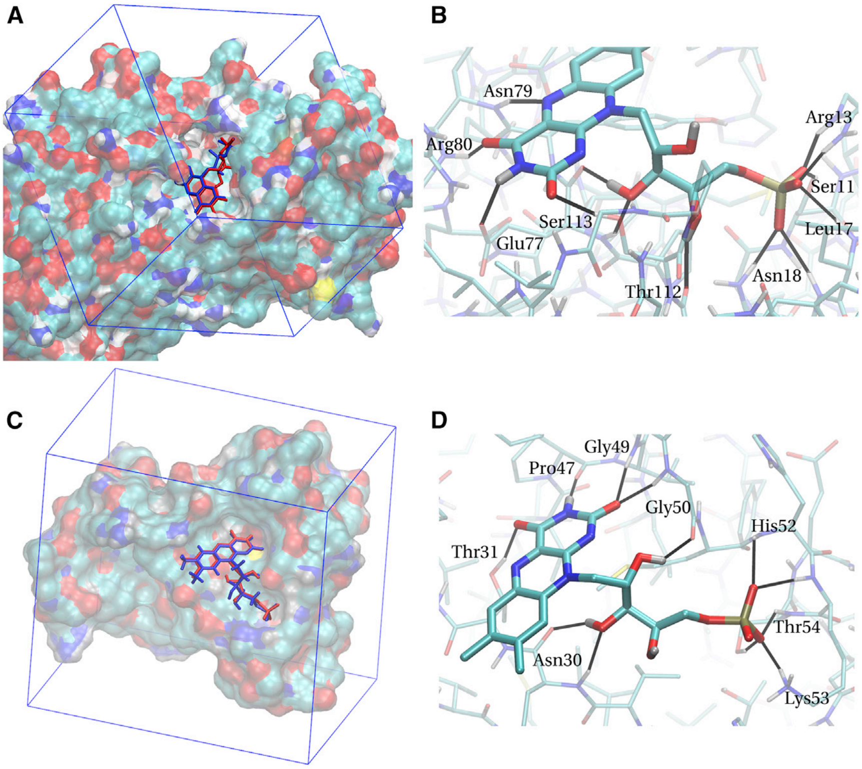

FIGURE 2 Redocking of FMN to two FMN-binding proteins. (*A*) Redocking to FerB from *Paracoccus denitrificans* (PDB: 3U7R) (21). (*Blue*) Experimental binding pose of FMN; (*red*) best pose obtained from computational redocking. (*Rectangular box*) Autodock search region. (*B*) Closeup of the redocked pose of FMN shown in (*A*), indicating individual hydrogen bonds (*black*) together with the protein residues involved. (*C*) Redocking to FMN-binding protein (FMN-bp) from *Desulfovibrio vulgaris* (Miyazaki F) (PDB: 1AXJ) (20); same color-code as in (*A*). (*D*) Closeup of the redocked pose of FMN shown in (*C*), indicating individual hydrogen bonds (*black*) together with the protein residues involved. To see this figure in color, go online.

runs with 7000 generations per run were carried out, with 75 individuals per run for FMN-bp and 100 individuals per run for FerB (to account for the larger search volume). The maximal number of energy evaluations was set to an arbitrarily high value (4×10^9) to enforce the number of generations as limiting criterion. The resultant 1200 docking poses for each protein were then clustered with a root-mean-square deviation (RMSD) cutoff of 3.0 Å. This means that all poses with an RMSD relative to the global lowest-energy pose that is smaller than the cutoff were included in the first cluster. The lowest-energy pose among the remaining poses was then the reference for the second cluster, and so on. This yielded large numbers of clusters in total (~50 for FMN-bp and almost 200 for FerB), of which only the lowest-free energy ones were significantly populated however.

FMN docking to MtrC in the SS state

The starting point was the crystal structure of MtrC as reported recently in Edwards et al. (11). It contains five Ca ions on the surface, which may originate from the buffer solution used for crystallization. We opted to remove four of the five Ca ions. The fifth one, next to heme 3 in the structure, appears to be a little more buried in the structure, which is why we opted to retain it. MtrC was chosen to be in the fully reduced state, assuming that this is the physiologically more relevant state if the cytochrome was to reduce a docked flavin. Test calculations in the SH state in fact only yielded a small impact of heme redox state on FMN affinity (see below and in the Supporting Material). Protonation states were chosen according to the results of the pK$_a$ estimator PropKa 3.1 (22). The crystal water was removed. The protein structure was then relaxed (energy-minimized) in NAMD 2.9 (32) using the AMBER03 force field (26) with heme parameters as in our previous studies (33–35). The relaxed structure was used for the dockings. As for the two proteins from the redockings, nonpolar hydrogens were merged in AutodockTools.

The blind docking was carried out similarly as for the FMN-binding proteins above. The same default Autodock atomic parameters and the same atomic charges for FMN were used as before, with atomic charges for protein atoms taken from the AMBER03 force field (26) and the ones for the heme cofactors and axial histidine ligands from previous work (34). Eight of the ten hemes in MtrC are solvent-accessible. Hemes 3 and 8 (see numbering in Fig. 1 *A*) are buried inside the protein and were thus not considered as viable docking targets. Thus, an individual blind docking procedure was carried out for each of the other eight hemes, 10, 9, 7, 6, 1, 2, 4, and 5. For each heme to which FMN was docked, a search box was centered on the heme with enough space in all directions to allow docking in the heme's extended surrounding. This yielded box-lengths in the range of 25–40 Å in each direction. For each heme, 1200 individual docking runs were carried out. The populations for the genetic algorithm were chosen between 75 and 125 individuals for each run depending on the box size. Each docking run consisted of 7000 generations in the genetic algorithm. The resultant 1200 poses were clustered with an RMSD cutoff of 3.0 Å resulting in ~100 clusters for each heme.

Molecular dynamics

Possible conformational changes upon cleavage of the disulphide bond were investigated with molecular dynamics (MD) simulations. As exploratory runs at room temperature or slightly elevated temperature showed no significant changes in the structure, SA was used to heat up the protein to higher temperatures and subsequently cool it down to room temperature. Before SA, the protein was equilibrated at room temperature. For this part of the study, all hemes were treated as oxidized corresponding to the conditions in the study of Edwards et al. (11); the same protonation states as in the dockings were used but all of the five calcium ions were included (as a nonstructural ion could now simply diffuse away during the dynamics). The protein was solvated with a water layer of thickness 15 Å and sodium and chloride ions were added to neutralize the system and obtain a salt concentration of ~0.1 M. The disulphide bond was treated as closed (SS state) initially. All of the subsequent MD simulations were carried out with NAMD 2.9 in periodic boundary conditions. The system was energy-minimized for 5000 steps before the solvent was equilibrated for 500 ps with the protein kept frozen, using periodic temperature rescaling to 300 K and a barostat with target pressure of 1 bar. Then the volume was fixed to the average value and the temperature rescaling was retained while the protein was slowly released by restraining it with successively weaker harmonic force constants of 99, 75, 50, 25, 10, 5, 1, 0.1, and 0.01 kcal/mol/Å^2. The duration of each protein equilibration step was 250 ps and the MD time step was 1 fs until 5 kcal/mol/Å^2, and these values increased to 500 ps and 2 fs thereafter. All restraints were then released, the barostat switched on again, and the thermostat changed to a Langevin thermostat. The system was equilibrated for 7 ns, after which the disulphide bond was cleaved and the two sulfur atoms were saturated with hydrogen atoms. In this SH state, the system was equilibrated for another 5 ns. The output of this last equilibration step served as input for the SA runs.

Simulated annealing

Simulated annealing (SA) is commonly used to accelerate the sampling of possible protein conformational changes (36–38). The annealing protocol typically consists of instantaneously heating the system to a very high temperature, several 100 K above room temperature, and stepwise cooling of the system down to room temperature. Test simulations at elevated temperature revealed that Domain III is surprisingly stable while the other domains, particularly Domain I, were less stable. It was found to be necessary to restrain certain protein regions during SA to avoid denaturation of the protein structure at high temperature. To exert restraints that were as mild as possible, the targeted MD feature in NAMD was used. This option allows one to restrain the total RMSD of a specified protein region with respect to a reference structure, rather than restraining atoms to individual reference positions. Two separate restraint regions were defined: one region comprising the backbone atoms of the entire Domain I, parts of Domains II and IV, and hemes 1, 2, 8, 9, and 10, and the other region containing the α-helix connecting Domains II and III. These regions are depicted in Fig. 3. They were chosen to keep the restraints clear from Domain III as much as possible. A force constant of 75 kcal/mol/Å^2 was used to harmonically restrain each region to zero RMSD with respect to its initial structure for the SA runs, which is the final structure of the equilibration at room temperature (see above).

The system with restraints applied as described above was simulated at the initial temperature for 1 ns, followed by cooling to 300 K by lowering the thermostat temperature every 100 ps in steps of 50 K. At 300 K, the simulation was run for 1 ns with the RMSD restraints active and then for another 1 ns with the restraints turned off. The initial temperatures were determined by investigating the protein behavior over a range of temperatures from 500 to 900 K. Potentially significant conformational changes were not found at $<$600 K. On the other hand, at temperatures $>$700 K, Domain III often showed a denaturated structure upon cooling. Hence, initial temperatures of 600, 650, and 700 K were chosen. Twenty-four SA runs with initial temperatures of 600 and 650 K and 28 SA runs at 700 K starting temperature were carried out. To isolate conformational features specific to the SH state, similar SA runs were carried out for the SS state for comparison, with 20 runs for each of the three temperatures. The final snapshot of one 700-K SA run was selected and further equilibrated for 110 ns at room temperature. As remarked in detail in the Results and Discussion, the combined SA and room temperature continuation resulted in a conformational change of the loop containing the two cysteines ("cys-loop" from now on), with the cys-loop making a large-scale motion toward Domain II, bringing the two now unbound cysteines almost 30 Å apart in the process. The conformational change observed was overall stable during this window of time, with no sign of return to the initial structure.

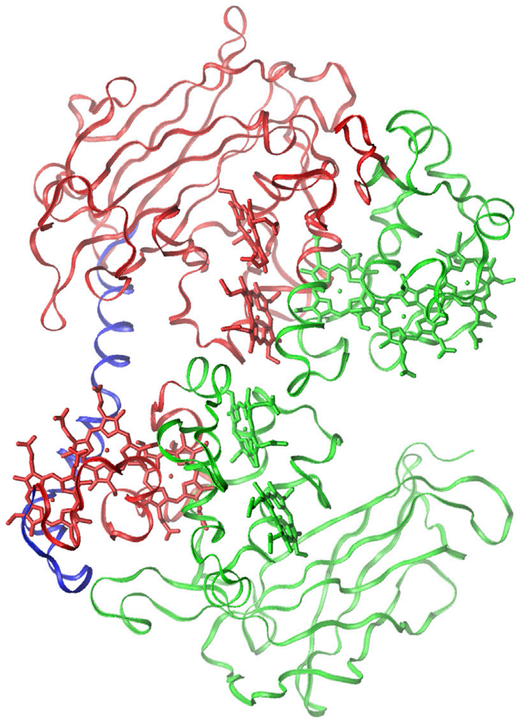

FIGURE 3 Setup for RMSD-restrained SA MD runs for MtrC. The two regions with RMSD restraints applied during the SA MD runs are depicted (*green* and *blue*, respectively). These were separately restrained to a target RMSD of 0 Å with respect to their initial structure, i.e., the final structure after equilibration at room temperature (see *SA protocol*). (*Red*) No restraints were applied to this region; it is allowed to move freely. To see this figure in color, go online.

Four randomly chosen snapshots after 60-ns run time and the final snapshot at 110 ns were selected for subsequent docking studies (see below).

FMN docking to annealed MtrC in the SH state

Several potential regions of interest arose due to the large-scale motion of the cys-loop: The top of the barrel where the loop had moved away; the front of the protein where the loop was making contact to Domain II; the region around heme 4 and 5 where the cys-loop now passed by; and the region around heme 7, which was suggested as a potential binding site by Edwards et al. (11). With the five snapshots chosen for docking, this resulted in 20 docking jobs in total, with box dimensions and genetic algorithm populations similar to the dockings in the SS state. To scan for potential new binding sites more rapidly, we only ran 300 runs for each snapshot and binding region (leaving the clustering RMSD cutoff at the default value of 2.0 Å for these dockings). For the dockings to hemes 4 and 7, we found one interesting snapshot each for which we carried out more extensive docking runs (1200 runs in total, clustering at 3.0 Å as for the SS state dockings). The hemes were chosen to be in the all-reduced state to facilitate comparison of binding affinities with docking runs carried out for the SS state. Additional dockings to hemes 4 and 7 were also carried out for the all-oxidized state, which was the experimental redox state in the disulphide cleavage experiment (11) and the state for which the SA simulations were carried out. We found that the docking results are rather insensitive to the exact heme redox state for a given protein configuration, with the main effect a slight increase in affinity (see the Supporting Material).

RESULTS AND DISCUSSION

Redocking to flavin-binding proteins

Histogram of clusters

The clusters obtained from docking were ordered according to the pose with lowest binding free energy in each cluster. In Fig. 4 we show the size of the clusters versus the lowest binding free energy of the poses in the cluster for both FMN-binding proteins. The corresponding Autodock output is provided in the Supporting Material. In both cases the first cluster containing the best pose with the overall lowest binding free energy is separated from the next cluster by a significant energy gap (2–3 kcal/mol). In the case of FerB (Fig. 4 *A*), the cluster containing the best pose is also the most populated one, with almost one in four individual docking runs (262:1200) resulting in a pose in that cluster. In the case of FMN-bp (Fig. 4 *B*), there are two other clusters with comparable population to the cluster with the best pose (one of them, the fourth cluster, even being a bit larger). However, these are already 3 kcal/mol higher in energy than the best overall pose.

The statistical significance of these results was investigated by dividing the total 1200 poses for FerB in eight independent groups and clustering the poses in each group independently with the default RMSD cutoff of 2.0 Å. All of these eight

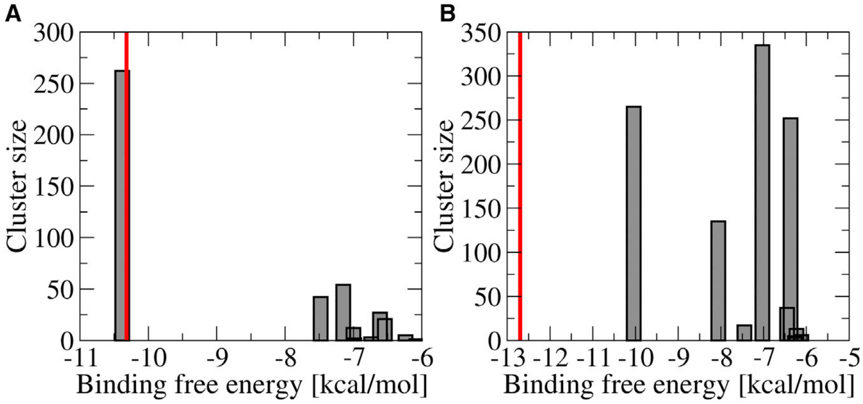

FIGURE 4 Histograms for redocking to two FMN-binding proteins (only the first 10 clusters are shown). (*A*) Redocking to FerB. (*B*) Redocking to FMN-bp. (*Red vertical bars*) Experimental binding free energies obtained from the experimental dissociation constants K_d (21,39). To see this figure in color, go online.

individual clusterings yielded lowest energy poses in close agreement with the best pose obtained by clustering all of the 1200 poses, and the corresponding eight histograms were similar to the overall histogram shown in Fig. 4.

RMSD

The best redocked FMN poses with the lowest binding free energy are shown in Fig. 2, *A* and *C*, for FerB and FMN-bp, respectively (structures in *red*). They are overlaid on the experimental crystal/NMR structure of FMN (shown in *blue*). Both experimental structures of the ligand are reproduced very well with RMSD values for the heavy atoms of FMN of 1.3 Å (FerB) and 1.1 Å (FMN-bp), respectively. Most of the residual deviation arises from the side chain, as the RMSD values for the headgroup alone amounts to 0.3 Å (FerB) and 0.5 Å (FMN-bp), respectively. Including both headgroup and phosphate tail still yields only 0.4 Å for FerB and a larger RMSD for FMN-bp of 1.0 Å. This indicates that the small deviations for FerB are mostly due to the flexible ribitol side chain and for FMN-bp due to the ribitol and phosphate groups.

Binding affinities

The ligand dissociation constants as obtained from Autodock's free energy force field are $K_d = 25$ nM and 43 nM for the best poses for FerB and FMN-bp, respectively. The value for FerB matches the experimental dissociation constant of 27 ± 2 nM very well (21) (indicated by a *red bar* in Fig. 4 *A*). The value for FMN-bp is somewhat higher than the experimental value, $K_d = 0.465$ nM (39), corresponding to an underestimation of binding affinity by 2.7 kcal/mol. However, it should be noted that in Kitamura et al. (39), the protein is likely to form a dimer in solution while the NMR structure used here for docking (PDB: 1AXJ) is monomeric. The presence of an additional protein subunit in the dimer could stabilize the bound FMN further and hence lower the binding free energy. Interestingly, when we intentionally excluded the experimental binding site from the search region in FMN-bp, the dissociation constant of the best pose increased from 43 nM to 770 μM and the histogram showed a continuum of clusters. This is a further strong indication that the protein has only one well-defined binding site.

Hydrogen bonding and ionic interactions

The specific interactions of FMN in the two binding sites of FerB and FMN-bp are shown in Fig. 2, *B* and *D*, respectively. It can be seen that the mold into which the flavin docks (illustrated in Fig. 2, *A* and *C*) provides in each case four hydrogen bonds for the flavin headgroup; from the five headgroup atoms capable of forming hydrogen bonds, three (FMN-bp) or four (FerB) do so. Together with a number of hydrogen bonds formed by side chain and phosphate tail, this yields in total 13 (FerB) and 12 (FMN-bp) hydrogen bonds, respectively. This result compares very favorably with a total number of 12 and 11 hydrogen bonds for the experimental structures for FerB and FMN-bp, respectively. In addition, each binding site features an ionic interaction with the phosphate tail of FMN (Arg^{13} and Lys^{53}, respectively). These features can be compared to the interactions observed in MtrC to rationalize the much weaker binding observed there (see below).

In summary, the results obtained here for two known FMN-binding proteins give credence to both the parameterization of ligand and protein and the blind docking protocol. The overall good agreement with experimental binding poses and binding affinities make us confident that the same protocol can give a faithful prediction of the interaction of FMN with other proteins such as MtrC.

Docking to MtrC in the SS state

Binding affinities

Preliminary tests showed that searching the entire protein surface at once was ineffective. Therefore, we decided to search a large region around each heme individually for possible docking sites, as described in the Materials and Methods. This approach is justified by recent NMR results (14) indicating that FMN binds closely to a heme. Of the eight hemes subjected to docking, six yielded FMN binding poses in (or almost in) van der Waals contact to the heme. Docking to the two central hemes 1 and 6 (see Fig. 1 *A* for heme labels) resulted in final poses that were closer to hemes 2 and 7, respectively. Hence, association of FMN with hemes 1 and 6 seems very unlikely. Table 1 summarizes K_d for the best poses for each heme. As can be seen, these range from 490 μM for heme 2 (*center left* in Fig. 1 *A*) to 30 mM for heme 5 (*top* in Fig. 1 *A*).

For comparison, we also docked FMN to a region of the protein surface in Domain I that is far away from any of the hemes, i.e., in a region that is expected to be functionally irrelevant for flavin docking. This yielded a dissociation constant of 2.9 mM for the lowest free energy pose (entry

TABLE 1 Dissociation constants K_d for each heme in MtrC as obtained from docking in the SS state

Heme	K_d (mM)
1	no hit
2	0.49
3	buried
4	12
5	29
6	no hit
7	7.4
8	buried
9	8.9
10	17
Protein surface	2.9

"No hit" means that the docking did not yield any ET-relevant poses. Hemes denoted as "buried" were not subjected to docking. For comparison, the lowest K_d for docking to a functionally not relevant part of the surface of MtrC is denoted by "protein surface".

"protein surface", *last row* in Table 1), a value similar to the 770 μM obtained for FMN-bp with the FMN binding region excluded during docking (see Redocking to Flavin-Binding Proteins). This suggests that dissociation constants on the order of 1 mM can easily be achieved on globular protein surfaces that do not contain specific FMN binding motifs. It further suggests that heme 2 is the only heme in MtrC to have an affinity for FMN that is stronger, but not much stronger, than the base-line affinity corresponding to 1 mM.

According to recent NMR measurements, the binding stoichiometry flavin/cytochrome = 1:1 (14). Our docking results are consistent with this experimental result. K_d for heme 2 is one order-of-magnitude lower than the K_d for the heme with the second highest affinity, and heme 2 is the only heme to exceed the aforementioned base-line affinity. Thus, it appears to be reasonable to conclude that the affinity of FMN to heme 2 accounts for a good fraction of the overall affinity of FMN to MtrC. This is further supported by the good agreement between the calculated dissociation constant for heme 2 (490 μM) and the experimental value of 255 $\pm$ 126 μM.

Binding poses

The structure of the FMN-heme 2 complex is less clearly defined. The histogram for heme 2 in Fig. 5 does not show a single most favorable cluster that is significantly lower in energy than the others, in contrast to what was found for redocking to FMN binding proteins (Fig. 4). Rather, the histogram shows an almost continuous spectrum of binding free energies, with the best pose only 0.1 kcal/mol lower in free energy than the second-best one and 0.4 and 0.6 kcal/mol lower than the two largest clusters. The lowest energy poses in the different clusters only agree in the location relative to heme 2, whereas they differ significantly in orientation. This is illustrated in Fig. 6 *A*, where the lowest energy poses from clusters 1 (overall lowest binding free energy), 4 and 6 (two largest clusters in the histogram), are shown. This is akin to the observations for docking to the protein surface excluding the binding site in FMN-bp and suggests that while heme 2 is the likely binding site for FMN, there is no single well-defined binding pose to heme 2.

The best docking pose for FMN docking to heme 2 in MtrC can be compared to the redocking poses obtained for FerB and FMN-bp (see Redocking to Flavin-Binding Proteins). Fig. 6 *B* shows the interaction network for FMN at heme 2. Comparing this docking site to the flavin-binding proteins (Fig. 2, *B* and *D*), several features become apparent: the number of hydrogen bonds formed between FMN and MtrC is only 8, compared to 12–13 hydrogen bonds in the flavin-binding proteins. The ionic interaction observed in FMN-bp and FerB is absent in MtrC: the positively charged protein side chain (Lys[241]) that does interact with FMN only participates in a hydrogen bond with the ribitol side chain. Inspecting the hydrogen-bonding network more closely, it can be seen that the headgroup is hardly at all involved in bonding: it merely forms one hydrogen bond, compared to the mold in FerB or FMN-bp that provides four hydrogen bonds in each case. This difference alone accounts for 60–75% of the additional hydrogen bonds in FerB and

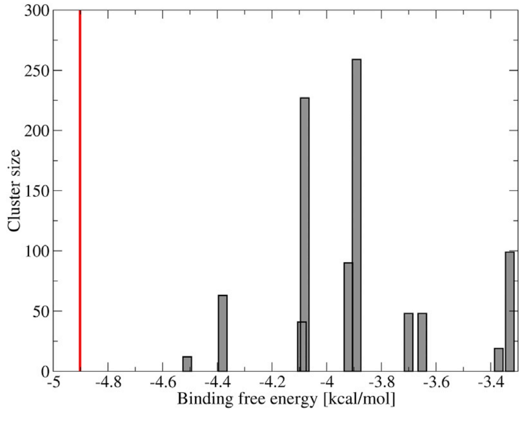

FIGURE 5 Histogram for docking of FMN to heme 2 in the crystal structure of MtrC (only the first 10 clusters are shown). (*Red vertical bar*) Experimental binding free energy obtained from the experimental dissociation constant K_d (14). To see this figure in color, go online.

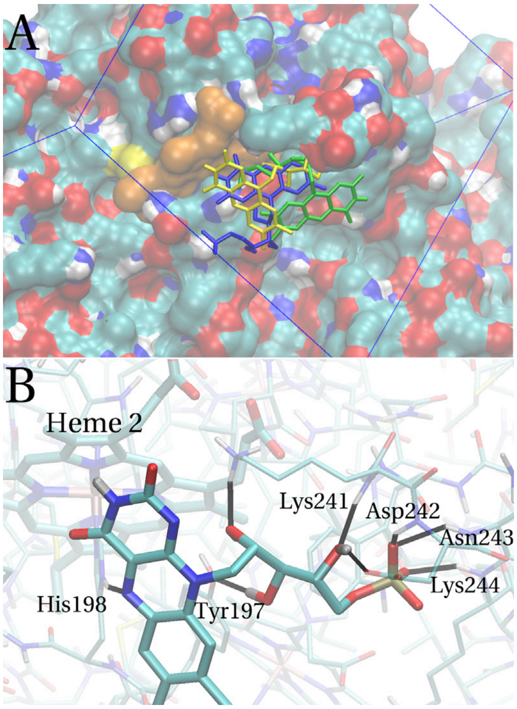

FIGURE 6 Docking of FMN to heme 2 in the crystal structure of MtrC. (*A*) Best poses of FMN in clusters 1 (*yellow*), 4 (*green*), and 6 (*blue*) of the histogram shown in Fig. 5 (i.e., the cluster with the strongest binding affinity and the two most populated clusters). Heme 2 is shown (*orange*) as well as the rectangular Autodock search box (*blue*). (*B*) Closeup on the best pose of FMN in cluster 1, indicating individual hydrogen bonds (*black*) together with the protein residues involved. The closest distance between the planar headgroup of FMN and the porphyrin edge is 3.5 Å. To see this figure in color, go online.

FMN-bp. These observations are consistent with the idea of the absence of a well-defined binding site in MtrC: the flexible ribitol side chain and phosphate tail can be expected to easily form hydrogen bonds on a globular protein surface while the requirements for the large rigid headgroup of FMN to form multiple hydrogen bonds (as in FerB and FMN-bp) are higher because that headgroup does not contain rotatable polar groups that could be easily positioned to interact with H-bond acceptors or donors. Similarly, positively charged protein side chains are generally far less abundant than potential hydrogen-bonding partners and hence, the absence of a positively charged lysine or arginine group at the right place to bind to the phosphate tail is in line with the lack of a well-defined binding site.

Conformational change in the SH state

Recent experiments suggest that FMN binding to MtrC is stronger when the disulphide bond is broken (the SH state) (11). Because no experimental structure is available for the SH state, we attempted to rationalize this finding by carrying out SA MD runs to probe the flexibility of the protein in this state. SA runs were carried out for both the SH and the SS states, which allow us to relate any differences observed to the cleavage of the disulphide bond. The simulation details are given in the Materials and Methods. We found that the front part of the loop containing the two cysteines (above heme 7, indicated in Fig. 1 *A*) shows some flexibility in both sets of simulations. Whereas in the SS state this section can only flip upwards, in the SH state the entire loop can lift off the barrel and move away even within the comparatively short nanosecond timescale of present SA MD runs.

Upon further MD simulation of one such structure for >100 ns at room temperature, we observe that the loop actually moves all the way to the front of the protein, somewhat inserting itself between Domain II to the left and some other loop of Domain III to the right, forming hydrogen bonds and hydrophobic interactions with residues from these loops. The backbone seemed somewhat stable over the last few tens of ns, suggesting that this could possibly be a stable structure. Fig. 7 *A* shows this final simulated structure of the SH state overlaid onto the crystal structure for the SS state; Fig. 7 *B* shows a closeup of the final position of the cys-loop. To further illustrate the magnitude of the conformational change observed, Fig. S1 in the Supporting Material shows the S-S distance of the two cysteines over the entire trajectory (SA and subsequent room temperature continuation). During the last few tens of ns, the distance reaches a level of 25–30 Å. Interestingly, the backbone of the barrel in Domain III is largely unaffected by the structural changes in the loop region and the switching motion of the loop to the front, as shown in Fig. 7 *A*. No significant structural changes could be observed in the vicinity of heme 7, which was hypothesized to be a possible binding site in the SH state (11).

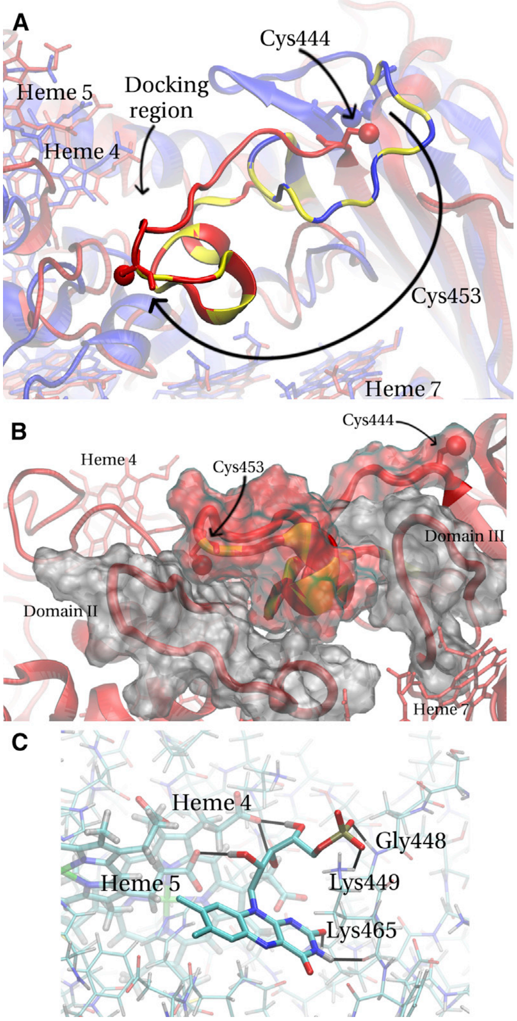

FIGURE 7 (*A*) Conformational switch of the cys-loop obtained after SA MD and subsequent 110 ns of room temperature MD in the SH state. Final structure obtained (*red*) and overlaid onto the crystal structure for the SS state (*blue*) (11). The two cysteines forming the disulphide bond and hemes 4, 5, and 7 are depicted (*licorice*) as well as the sulfur atoms (*van der Waals spheres*). For ease of comparison, the front part of the loop (according to the crystal structure position) is highlighted for the SH (*yellow-red*) and SS (*yellow-blue stripes*) states, respectively. (*Long arrow* for Cys[453]) Conformational switch upon cleavage of the disulphide bond. Upon cleavage of the disulphide bond between Cys[444] and Cys[453], Cys[444] remains rather stationary while the loop containing Cys[453] swings over to the front, translocating Cys[453] by ~25 Å. (*Docking region*) Region where FMN was docked after SA MD (refer to *C*). (*B*) Closeup of the final position of the cys-loop in the SH state. The color-code is the same as in (*A*). In addition, loops belonging to Domains II and III, respectively, are shown. These loops, as well as the cys-loop, are drawn also in surface representation to illustrate their spatial extension (*red* for the cys-loop; *silver* for the loops from Domains II and III). It can be seen that the cys-loop containing Cys[453] fills to a large extent the gap between the two loops shown (in *gray*). (*C*) Docking of FMN to the region around heme 4 for a structure in the SH state (similar to the one shown in *red* in *A*). (*Black lines*) Hydrogen bonds. It can be seen that the flavin simultaneously interacts with the propionates of hemes 4 and 5 and with backbone and side-chain atoms of the translocated cys-loop. To see this figure in color, go online.

Docking to MtrC in the SH state

As outlined in the Materials and Methods, we also docked FMN to several snapshots from the SA trajectories featuring the conformational switch of the cys-loop. As described in more detail in the Supporting Material, we arrived at one potentially relevant binding pose shown in Fig. 7 *C* with the flavin close to heme 4 and 5 (7.1 and 5.2 Å edge-to-edge distance, thus relevant for ET). While this pose does not yield an affinity better than 2 mM (weaker than the best pose at heme 2 in the SS state), it does show several features that we would like to outline here, as they suggest the potential formation of an actual binding site here. (See the Supporting Material for the complete discussion.)

The pose shown in Fig. 7 *C* forms seven hydrogen bonds as well as an ionic bond. Three hydrogen bonds are formed with proprionates of heme 4 and 5 (left of the flavin) and four with residues from the cys-loop (right of the flavin), which after the conformational switch passes by in the vicinity of heme 4 (see also Fig. 7 *A*). Two hydrogen bonds are formed by the flavin headgroup, which also enters some kind of cleft, in contrast to the pose lying on the surface for heme 2 in the SS state. (The cluster histogram also shows a preference for the pose shown in Fig. 7 *C*, unlike the unclear picture observed for heme 2 (see Figs. 5 and 6)). Thus, different protein regions (the hemes 4 and 5 and the cys-loop) come together by the conformational switch to provide hydrogen bonds and ionic interactions for the flavin from both sides, which might be the onset of an actual binding site. In the search for a binding site of FMN in the SH state that explains the strong affinity observed in experiment (11), this seems like the most interesting starting point to us. (We would like to mention that we could not find any ET-relevant binding poses in the vicinity of heme 7.)

A binding site at heme 4 would be particularly interesting in the light of our previous redox potential calculations on the homolog MtrF (15) that revealed this heme to have the lowest redox potential (at ~−0.27 V), even lower than the redox potential of free FMN (−0.22 V) (40) and hence a potentially more efficient electron donor to FMN than the other hemes higher in potential. The redox potential was decreased with respect to the other hemes in particular by a neighboring propionate hydrogen bonding to one of its histidines, something that we observe for heme 4 in MtrC as well. Thus, the observed conformational change of the cys-loop in the SH state could possibly create a binding site close to the energetically most favorable heme.

CONCLUSIONS

In this work, we investigated docking of FMN to the solvent-accessible hemes in the deca-heme cytochrome MtrC, as well as possible conformational changes upon cleaving a disulphide bond. At first we validated the blind docking protocol used by redocking of FMN to two flavin-binding proteins and found that both the experimental structures of the FMN-protein complexes as well as the binding affinities could be reproduced in very good agreement with experiment. The protocol, originally introduced by Hetényi and van der Spoel (18,19) and further tested in this work for FMN binding, is generally applicable and appears to be a successful docking strategy in situations where multiple putative docking sites exist.

Application of the blind docking protocol to FMN binding to MtrC shows that the interactions with this protein are much weaker than with the flavin-binding proteins. We did not find a well-defined binding site in MtrC. Our docking studies indicate that interaction with heme 2 is strongest with a K_d of 490 μM, in close agreement with the experimental value of 255 μM. A possible qualitative explanation for the relatively weak binding would be the limited number of hydrogen bonds that the planar headgroup of FMN can form with MtrC, and the absence of ionic interactions. In the lowest free energy pose obtained, only one hydrogen bond is formed between the headgroup and MtrC, whereas four strong hydrogen bonds are formed with the FMN-binding proteins. Overall, the relatively weak and reversible binding of MtrC seems to be consistent with the role of a redox shuttle: binding to heme 2 in MtrC is stronger than on the protein surface but still relatively weak, so that after ET, rapid unbinding is possible. This presumes, however, that the interaction with of the reduced flavin with MtrC is similarly weak, which we have not further investigated in this study.

Our results are consistent with previous docking studies on MtrC homologs, investigating the interaction of FMN with the deca-heme protein OmcA and of a related redox molecule, Anthraquinone 2,6-disulfonate, with the undeca-heme protein UndA (14). In both cases heme 2 was identified as the preferred docking site, similar to this result for MtrC. This begs the question whether there is any functional relevance for interaction with this particular heme. The microscopic redox potential computed for heme 2 in the homolog MtrF was at the higher end among the 10 hemes, −0.06 V (15), implying that electron transfer to FMN ($\epsilon^0 = -0.22$ V) is possible but would be slightly uphill if specific interactions between protein and FMN are neglected.

Prompted by the recent suggestion that reduction of the disulphide bond in MtrC (SH state) strongly increases FMN binding, we investigated possible conformational switches upon cleavage of this bond via SA MD. We found a major conformational change of the loop containing the disulphide group upon cleavage of the bond. While we could not (yet) establish a new binding site featuring an affinity in agreement with the experimentally observed stable binding, we could identify a potential binding site in the vicinity of heme 4—one of the hemes suggested to have a redox potential lower than that of FMN itself (15,40), with corresponding implications for ET functionality.

SUPPORTING MATERIAL

Supporting Materials and Methods, one figure, and one table are available at http://www.biophysj.org/biophysj/supplemental/S0006-3495(15)01114-5.

AUTHOR CONTRIBUTIONS

J.B. and K.M.R. designed the research; M.B. performed the research; M.B. contributed new reagents/analytic tools; M.B. and J.B. analyzed data; and M.B., K.M.R., and J.B. wrote the article.

ACKNOWLEDGMENTS

We thank Dr. Adam Kubas for help with the RESP charge parameterization of FMN.

M.B. gratefully acknowledges an IMPACT studentship cosponsored by University College London and Pacific Northwest National Laboratory through the U.S. Department of Energy Office of Biological and Environmental Research Subsurface Biogeochemistry Research Science Focus Area program at the Pacific Northwest National Laboratory, the latter of which provided support for K.M.R. J.B. acknowledges support from the Royal Society via a University Research Fellowship and from the Engineering and Physical Sciences Research Council via grant No. EP/M001946/1. MD simulations were carried out on ARCHER, the UK National High Performance Computing facility (Edinburgh, Scotland), to which access was granted via the Materials Chemistry Consortium (Engineering and Physical Sciences Research Council grant No. EP/L000202).

REFERENCES

1. Shi, L., T. C. Squier, …, J. K. Fredrickson. 2007. Respiration of metal (hydr)oxides by *Shewanella* and Geobacter: a key role for multihaem *c*-type cytochromes. *Mol. Microbiol.* 65:12–20.
2. Richardson, D. J., J. N. Butt, …, T. A. Clarke. 2012. The ‘porin-cytochrome’ model for microbe-to-mineral electron transfer. *Mol. Microbiol.* 85:201–212.
3. Blumberger, J. 2015. Recent advances in the theory and molecular simulation of biological electron transfer reactions. *Chem. Rev.* 115:11191.
4. White, G. F., Z. Shi, …, T. A. Clarke. 2013. Rapid electron exchange between surface-exposed bacterial cytochromes and Fe(III) minerals. *Proc. Natl. Acad. Sci. USA.* 110:6346–6351.
5. Brutinel, E. D., and J. A. Gralnick. 2012. Shuttling happens: soluble flavin mediators of extracellular electron transfer in *Shewanella*. *Appl. Microbiol. Biotechnol.* 93:41–48.
6. Pirbadian, S., S. E. Barchinger, …, M. Y. El-Naggar. 2014. *Shewanella oneidensis* MR-1 nanowires are outer membrane and periplasmic extensions of the extracellular electron transport components. *Proc. Natl. Acad. Sci. USA.* 111:12883–12888.
7. Marsili, E., D. B. Baron, …, D. R. Bond. 2008. *Shewanella* secretes flavins that mediate extracellular electron transfer. *Proc. Natl. Acad. Sci. USA.* 105:3968–3973.
8. von Canstein, H., J. Ogawa, …, J. R. Lloyd. 2008. Secretion of flavins by *Shewanella* species and their role in extracellular electron transfer. *Appl. Environ. Microbiol.* 74:615–623.
9. Coursolle, D., D. B. Baron, …, J. A. Gralnick. 2010. The Mtr respiratory pathway is essential for reducing flavins and electrodes in *Shewanella oneidensis*. *J. Bacteriol.* 192:467–474.
10. Clarke, T. A., M. J. Edwards, …, D. J. Richardson. 2011. Structure of a bacterial cell surface decaheme electron conduit. *Proc. Natl. Acad. Sci. USA.* 108:9384–9389.
11. Edwards, M. J., G. F. White, …, T. A. Clarke. 2015. Redox linked flavin sites in extracellular decaheme proteins involved in microbe-mineral electron transfer. *Sci. Rep.* 5:11677.
12. Edwards, M. J., A. Hall, …, T. A. Clarke. 2012. The crystal structure of the extracellular 11-heme cytochrome UndA reveals a conserved 10-heme motif and defined binding site for soluble iron chelates. *Structure.* 20:1275–1284.
13. Okamoto, A., K. Hashimoto, …, R. Nakamura. 2013. Rate enhancement of bacterial extracellular electron transport involves bound flavin semiquinones. *Proc. Natl. Acad. Sci. USA.* 110:7856–7861.
14. Paquete, C. M., and R. O. Louro. 2014. Unveiling the details of electron transfer in multicenter redox proteins. *Acc. Chem. Res.* 47:56–65.
15. Breuer, M., P. Zarzycki, …, K. M. Rosso. 2012. Thermodynamics of electron flow in the bacterial deca-heme cytochrome MtrF. *J. Am. Chem. Soc.* 134:9868–9871.
16. Breuer, M., P. Zarzycki, …, K. M. Rosso. 2012. Molecular structure and free energy landscape for electron transport in the decahaem cytochrome MtrF. *Biochem. Soc. Trans.* 40:1198–1203.
17. Breuer, M., K. M. Rosso, and J. Blumberger. 2014. Electron flow in multiheme bacterial cytochromes is a balancing act between heme electronic interaction and redox potentials. *Proc. Natl. Acad. Sci. USA.* 111:611–616.
18. Hetényi, C., and D. van der Spoel. 2002. Efficient docking of peptides to proteins without prior knowledge of the binding site. *Protein Sci.* 11:1729–1737.
19. Hetényi, C., and D. van der Spoel. 2006. Blind docking of drug-sized compounds to proteins with up to a thousand residues. *FEBS Lett.* 580:1447–1450.
20. Liepinsh, E., M. Kitamura, …, G. Otting. 1997. Pathway of chymotrypsin evolution suggested by the structure of the FMN-binding protein from *Desulfovibrio vulgaris* (Miyazaki F). *Nat. Struct. Biol.* 4:975–979.
21. Sedláček, V., T. Klumpler, …, I. Kučera. 2014. The structural and functional basis of catalysis mediated by NAD(P)H:acceptor Oxidoreductase (FerB) of *Paracoccus denitrificans*. *PLoS One.* 9:e96262.
22. Sondergaard, C. R., M. H. M. Olsson, …, J. H. Jensen. 2011. Improved treatment of ligands and coupling effects in empirical calculation and rationalization of pK_a values. *J. Chem. Theory Comput.* 7:2284.
23. Morris, G. M., R. Huey, …, A. J. Olson. 2009. AutoDock4 and AutoDockTools4: automated docking with selective receptor flexibility. *J. Comput. Chem.* 30:2785–2791.
24. Morris, G. M., D. S. Goodsell, …, A. J. Olson. 1998. Automated docking using a Lamarckian genetic algorithm and an empirical binding free energy function. *J. Comput. Chem.* 19:1639.
25. Huey, R., G. M. Morris, …, D. S. Goodsell. 2007. A semiempirical free energy force field with charge-based desolvation. *J. Comput. Chem.* 28:1145–1152.
26. Duan, Y., C. Wu, …, P. Kollman. 2003. A point-charge force field for molecular mechanics simulations of proteins based on condensed-phase quantum mechanical calculations. *J. Comput. Chem.* 24:1999–2012.
27. Bayly, C. I., P. Cieplak, …, P. A. Kollman. 1993. A well-behaved electrostatic potential based method using charge restraints for deriving atomic charges: the RESP model. *J. Phys. Chem.* 97:10269.
28. Valiev, M., E. J. Bylaska, …, W. A. d. Jong. 2010. NWCHEM: a comprehensive and scalable open-source solution for large scale molecular simulations. *Comput. Phys. Commun.* 181:1477–1489.
29. Becke, A. 1993. Density functional thermochemistry. III. The role of exact exchange. *J. Chem. Phys.* 98:5648.
30. Lee, C., W. Yang, and R. G. Parr. 1988. Development of the Colle-Salvetti correlation-energy formula into a functional of the electron density. *Phys. Rev. B Condens. Matter.* 37:785–789.
31. Klamt, A., and G. Schuurmann. 1993. Cosmo: a new approach to dielectric screening in solvents with explicit expressions for the screening energy and its gradient. *J. Chem. Soc. Perkin Trans.* 2:799.

32. Phillips, J. C., R. Braun, …, K. Schulten. 2005. Scalable molecular dynamics with NAMD. *J. Comput. Chem.* 26:1781–1802.

33. Blumberger, J. 2008. Free energies for biological electron transfer from QM/MM calculation: method, application and critical assessment. *Phys. Chem. Chem. Phys.* 10:5651–5667.

34. Tipmanee, V., H. Oberhofer, …, J. Blumberger. 2010. Prediction of reorganization free energies for biological electron transfer: a comparative study of Ru-modified cytochromes and a 4-helix bundle protein. *J. Am. Chem. Soc.* 132:17032–17040.

35. Tipmanee, V., and J. Blumberger. 2012. Kinetics of the terminal electron transfer step in cytochrome *c* oxidase. *J. Phys. Chem. B.* 116:1876–1883.

36. Xu, D., M. Sheves, and K. Schulten. 1995. Molecular dynamics study of the M412 intermediate of bacteriorhodopsin. *Biophys. J.* 69:2745–2760.

37. Wriggers, W., and K. Schulten. 1998. Nucleotide-dependent movements of the kinesin motor domain predicted by simulated annealing. *Biophys. J.* 75:646–661.

38. Knecht, V., and H. Grubmüller. 2003. Mechanical coupling via the membrane fusion SNARE protein syntaxin 1A: a molecular dynamics study. *Biophys. J.* 84:1527–1547.

39. Kitamura, M., K. Terakawa, …, Y. Higuchi. 2007. Determination of the role of the Carboxyl-terminal leucine-122 in FMN-binding protein by mutational and structural analysis. *J. Biochem.* 141:459–468.

40. Brown, J. P. 1981. Reduction of polymeric azo and nitro dyes by intestinal bacteria. *Appl. Environ. Microbiol.* 41:1283–1286.

41. Breuer, M., K. M. Rosso, J. Blumberger, and J. N. Butt. 2015. Multiheme cytochromes in shewanella oneidensis mr-1: structures, functions and opportuntities. *J. R. Soc. Interface.* 12:20141117.

Flavin binding to the deca-heme cytochrome MtrC: Insights from computational molecular simulation

M. Breuer, K. M. Rosso, J. Blumberger

October 20, 2015

Supporting Information

1 Docking to MtrC in SH state

In the following we would like to discuss our docking studies to MtrC in the SH state in more detail. As outlined in Section 2 in the main text, four docking regions were chosen for docking FMN in the SH state. This was motivated by the observed large switching motion of the loop: This not only changed the structure around the area of the original disulphide bond (i. e. around Cys444, see Figure 7A in the main text) but also led to the localization of Cys453 and its adjacent residues at the front of the protein as illustrated in Figure 7B, main text. Thus, both of these areas were regions of interest. In addition, we noticed that the loop segment between the two cysteines (residues 445 to 452) now passed by in the proximity of heme 4 and 5. These were the only hemes whose local environment was visibly changed by the conformational switch and thus this was a region of particular interest for docking. Finally, we also included the region around heme 7 which had been suggested in experiment as a binding site in the SH state.[1]

The initial scans of 300 runs for all 20 dockings (the above four regions for five different snapshots) yielded no affinities stronger than the 490 μM found for heme 2 in the SS state, nor any other interesting features in most cases. However, we decided to investigate two dockings further, one for heme 4 and one for heme 7. The docking for heme 7 was studied further as it had shown the overall strongest affinity (760 μM); and the docking for heme 4, while having shown a weaker affinity of 2.2 mM, yielded a docking site that had actually formed between the proprionates of hemes 4 and 5 on the one hand and different residues of the cys-loop on the other hand - the structure presented in Figure 7C in the main text. Hence we carried out another 900 runs in each case, subsequently clustering the 1200 runs in total at an RMSD cutoff of 3.0 Å as for the SS dockings. In the following we discuss the results for both of the hemes.

For heme 4, the clustering of all 1200 runs at 3.0 Å revealed two best poses of equal affinity (both 2.2 mM): The aforementioned pose and a similar pose where the flavin head group is rotated a bit. In a coarser reclustering at 4.0 Å these two clusters actually merge and yield a large first cluster (116/1200 runs) which is succeeded in size only by a cluster worse in binding free energy by 0.63 kcal/mol (containing 168 runs). This stands in contrast to the observations for docking to heme 2 in the SS state which yielded a much more ambiguous picture with the energetically best cluster containing very few runs only (see the histogram in Figure 5; the qualitative features do not change when reclustering at 4.0 Å). This suggests that this best cluster obtained by docking to a snapshot from the annealing trajectory is more significant (compared to the other runs from that docking) than the best pose shown for heme 2 in Figure 6B. (Albeit the picture is still not nearly as clear as for the FMN-binding protein redockings in Figure 4.) In the following, we will inspect this binding pose more closely. Figure 7C in the main text shows the close-up of this best cluster and the hydrogen bonds involved.

While this FMN pose forms only seven hydrogen bonds (compared to eight for the best pose at heme 2 in the SS state), two of them are formed by the head group, involving H-bond donors from different directions: The

amino group of Lys465 and the backbone of Lys449, both part to the cys-loop. Lys465 actually forms an ionic bond with a proprionate of heme 4 which appears to be quite stable. (Suggesting that the proximity and hence availability of this lysine is not just coincidence.) The interactions of the tail involve three hydrogen bonds of the hydroxy-hydrogens to the proprionates of hemes 4 and 5; and an ionic interaction between the phosphate and Lys449, as well as hydrogen bonds between the phosphate and the same lysine and the backbone of Gly448 (also part of the cys-loop). Thus, while the tail can always be expected to find some hydrogen bonding partners (see our "negative test" docking to a random region at the bottom of Domain I), in this case the interactions happen with two different parts of the protein: The hemes from Domain II on the left side and different residues from the cys-loop on the right, which in particular hydrogen-bonds with two of the four potential hydrogen-bonding sites in the head group. The edge-to-edge distances of this pose are 5.2 Å to heme 5 and 7.1 Å to heme 4, in principle still close enough for relevant ET rates. It should also be noted that this is just one out of five snapshots investigated from the annealing trajectory; chances are that the protein has still not found its final structure after 110 ns or that induced fit effects might yield stronger actual interaction with the docked flavin. (Apart from the obvious possibility of Lys449, which ion-binds the phosphate, optimizing its conformation, further hydrogen bonds might be formed between one of the remaining potential acceptors in the flavin head group and some other backbone hydrogen from the cys-loop.) Hence, while we could not find a binding site yet that would yield affinities in agreement with the stable binding observed by Edwards et al.,[1] we would argue that the pose we found close to hemes 4 and 5 suggests a potential binding site as: the flavin head group actually enters some form of cleft, rather than just lying somewhere on the protein surface; in doing so, it forms several hydrogen bonds with comparatively buried partners; the tail simultaneously interacts with both sides of the cleft - the hemes on the left and the cys-loop on the right/top; and the formation of this site depends on the conformational change we observed, that brought the cys-loop into the position to form this cleft. Further investigations could take off from this tentative binding pose and aim at identifying an actually strong binding site.

For heme 7, we obtained a strongest affinity of 530 μM over the 1200 runs and the best pose was clearly separated from all following clusters by an energy difference of -1.21 kcal/mol. However, with an edge-to-edge distance to heme 7 of 9.9 Å, this binding pose seems hardly relevant for ET. In addition, even though this pose did form two hydrogen bonds involving the head group, all interacting protein residues were already closeby and to some degree solvent-exposed in the crystal structure, and none of them was part of the cys-loop; therefore, the occurence of this docking site does not seem connected to the conformational change of the cys-loop.

As an aside, in order to investigate the impact of the redox state, we repeated these 1200 dockings for both heme 4 and 7 with all heme charges set to oxidized and clustered again at 3.0 Å RMSD cutoff. **(While the simulated annealing had been carried out in the all-oxidized state to match the experimental redox state,[1] all dockings described above were carried out in the all-reduced state for comparison with**

results in the SS state; see Materials and Methods in the main text.) For heme 4, the first two poses were qualitatively unchanged and their binding free energies merely changed by -0.4 and -0.3 kcal/mol, respectively. We checked the next few poses and could also find them (with small changes) among the first clusters of the previous all-reduced dockings, suggesting that the same general poses were found and mainly changed their energetical ordering. The observations were similar for heme 7 where the best pose showed minor changes and changed in binding free energy by -0.5 kcal/mol; and the next few poses obtained with oxidized hemes could also be found among the poses obtained with the reduced heme charges, again with different ordering. These results suggest that our docking results are rather insensitive to the exact heme redox state (for a given protein configuration), with the main effect a slight increase in affinity.

Table S1: RESP charges used for flavin mononucleotide (FMN) as obtained with the B3LYP functional[2,3] and cc-pVTZ basis set, combined with the COSMO continuum solvation model[4] **using a relative permittivity of 4.0**.

Atom name	Charge [e]	Atom name	Charge [e]
N1	-0.396715	C5’	0.069370
C2	0.373383	O5’	-0.411130
O2	-0.560608	P	0.923193
N3	-0.040698	O1P	-0.763397
C4	0.149203	O2P	-0.763397
O4	-0.517263	O3P	-0.763397
C4A	0.590519	H3	0.237377
N5	-0.544057	H6	0.125184
C5A	0.162506	H7M1	0.083170
C6	-0.134921	H7M2	0.083170
C7	0.029809	H7M3	0.083170
C7M	-0.240263	H8M1	0.047064
C8	0.069789	H8M2	0.047064
C8M	-0.081776	H8M3	0.047064
C9	-0.180532	H9	0.116942
C9A	0.016667	H1’1	0.037677
N10	0.076287	H1’2	0.037677
C10	0.050082	H2’	0.098083
C1’	0.091311	HO2	0.357665
C2’	-0.022327	H3’	0.074538
O2’	-0.542320	HO3	0.253008
C3’	0.015111	H4’	0.115456
O3’	-0.366671	HO4	0.380892
C4’	0.051154	H5’1	0.031974
O4’	-0.628058	H5’2	0.031974

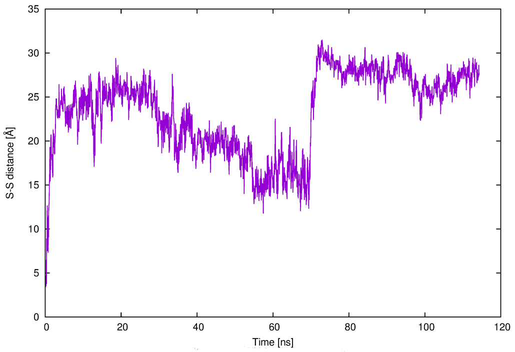

Figure S1: The S-S distance between Cys444 and Cys453 over the course of Simulated Annealing and subsequent dynamics at room temperature.

References

[1] Edwards, M. J., G. F. White, M. Norman, A. Tome-Fernandez, E. Ainsworth, L. Shi, J. K. Fredrickson, J. M. Zachara, J. N. Butt, D. J. Richardson, and T. A. Clarke. 2015. Redox linked flavin sites in extracellular decaheme proteins involved in microbe-mineral electron transfer. *Sci. Rep.* 5:11677.

[2] Becke, A. 1993. Density-functional thermochemistry. iii. the role of exact exchange. *J. Chem. Phys.* 98:5648.

[3] Lee, C., W. Yang, and R. Parr. 1988. Development of the colle-salvetti correlation-energy formula into a functional of the electron density. *Phys. Rev. B.* 37:785.

[4] Klamt, A., and G. Schuurmann. 1993. Cosmo: a new approach to dielectric screening in solvents with explicit expressions for the screening energy and its gradient. *J. Chem. Soc. Perkin Trans.* 2:799.
